# ECM stiffness affects cargo sorting into MSC-EVs to regulate their secretion and uptake behaviors

**DOI:** 10.1186/s12951-024-02411-w

**Published:** 2024-03-21

**Authors:** Zhixiao Liu, Yingying Liu, Yu Li, Sha Xu, Yang Wang, Yuruchen Zhu, Chu Jiang, Kaizhe Wang, Yinan Zhang, Yue Wang

**Affiliations:** 1 Department of Histology and Embryology, College of Basic Medicine, Naval Medical University, Shanghai, 200433 China; 2 School of Chemistry and Chemical Engineering, Center for Transformative Molecules, Zhangjiang Institute for Advanced Study and National Center for Translational Medicine (Shanghai), Shanghai Jiao Tong University, Shanghai, 200240 China; 3 Stem Cell and Regeneration Medicine Institute, Research Center of Translational Medicine, Naval Medical University, Shanghai, 200433 China; 4 Shanghai Institute of Stem Cell Research and Clinical Translation, Shanghai, 200120 China; 5 Shanghai General Hospital of Nanjing Medical University, Shanghai, 200086 China; 6 College of Basic Medicine, Naval Medical University, Shanghai, 200433 China; 7 School of Chemical Science and Engineering, Tongji University, Shanghai, 200092 China; 8 Ningbo Key Laboratory of Biomedical Imaging Probe Materials and Technology, Ningbo Cixi Institute of Biomedical Engineering, Ningbo Institute of Materials Technology and Engineering, Chinese Academy of Sciences, Ningbo, 315300 China; 9 Shanghai Key Laboratory of Cell Engineering, Shanghai, China

**Keywords:** Extracellular matrix, Stiffness, Mesenchymal stem cells, Extracellular vesicles, Drug delivery

## Abstract

Mesenchymal stem cell-derived extracellular vesicles (MSC-EVs) have garnered extensive attention as natural product-based nanomedicines and potential drug delivery vehicles. However, the specific mechanism for regulating MSC-EVs secretion and delivery remains unclear. Here, we demonstrate that extracellular matrix (ECM) stiffness regulates the secretion and delivery of EVs by affecting MSCs' cargo sorting mechanically. Using multi-omics analysis, we found that a decrease in ECM stiffness impeded the sorting of vesicular transport-related proteins and autophagy-related lipids into MSC-EVs, impairing their secretion and subsequent uptake by macrophages. Hence, MSC-EVs with different secretion and uptake behaviors can be produced by changing the stiffness of culture substrates. This study provides new insights into MSC-EV biology and establishes a connection between MSC-EV behaviors and ECM from a biophysical perspective, providing a basis for the rational design of biomedical materials.

**Graphical Abstract:**

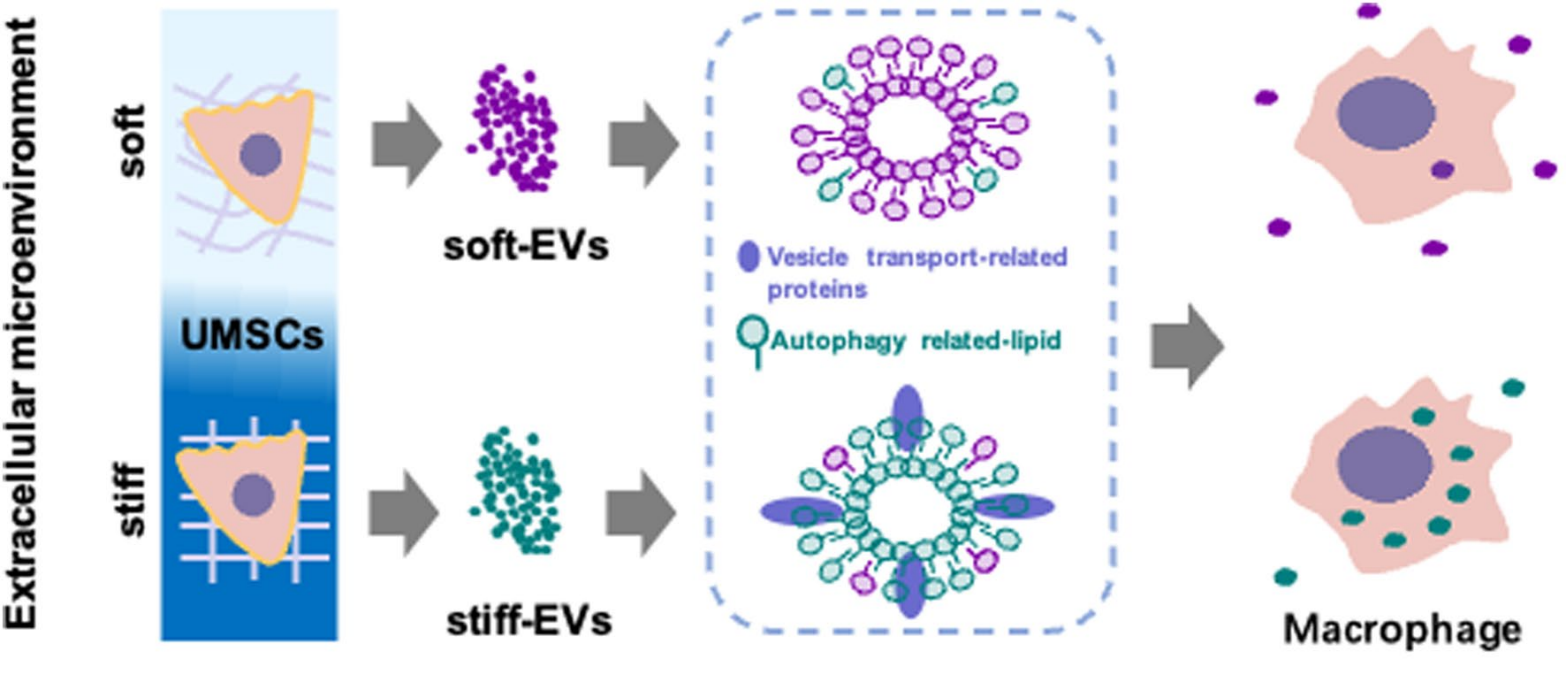

**Supplementary Information:**

The online version contains supplementary material available at 10.1186/s12951-024-02411-w.

## Introduction

Mesenchymal stem cells (MSCs) offer immense potential in treating various diseases and injuries due to their regenerative and immunomodulatory capabilities [1, 2]. However, using these cells in clinical applications has been challenging due to their high heterogeneity, limited availability, difficulty in preferential delivery to specific cells, and limited survival in vivo [3]. It has been discovered that extracellular vesicles (EVs) derived from MSCs can act as cell-free coordinators to transport functional cargos including lipids, proteins, and RNAs between cells to facilitate communication [4, 5]. MSC-EVs can target macrophages that are crucial in both innate and adaptive immune responses [6, 7]. Furthermore, MSC-EVs have shown potential as drug transport platforms for targeted delivery of exogenous cargo to specific cell types or tissues [8–12]. However, due to the evolutionarily complex biological properties of MSC-EVs, it remains hard to figure out how MSC-EVs sort and deliver their cargo to specific cells [13–16]. Understanding the regulatory mechanisms of MSC-EVs behaviors is crucial for prescribing their use in biomedicine.

Extracellular matrix (ECM) is a complex network of proteins and carbohydrates surrounding cells. Other than acting as biochemical support, it also provides physical cues that influence cellular behaviors including adhesion, migration, and differentiation [17–22]. Particularly, the mechanical properties of ECM have a profound impact on EV biology. For instance, a rigid ECM can promote EV secretion from tumor cells and increase tumor growth [23, 24]. In MSC studies, EVs obtained by 3D culture have higher amounts of anti-inflammatory and anti-apoptotic factors than 2D culture EVs [25]. Additionally, MSCs secrete 10 times more EVs on a soft matrix than on a rigid one, leading to a better repair effect on acute lung injury [26]. Nevertheless, how ECM induces these MSC-EVs responses is yet unclear.

We have previously established a scheme that uses cell substrates of varying stiffness to study the responses of cellular metastasis patterns to mechanical environments. In this study, we show that ECM stiffness can regulate the sorting of functional cargos into MSC-EVs, thus affecting their secretion and uptake by macrophages. Using multi-omics analysis, we found that ECM of lower stiffness hindered the sorting of vesicular transport-related proteins and autophagy-related lipids into MSC-EVs, suppressing their secretion and uptake by macrophages. Therefore, MSC-EVs of different phenotypes can be rationally made by varying the ECM stiffness. This study opens up new avenues to reveal the links between MSC-EV behaviors and mechanical environments, contributing to clarifying the mechanism of MSC-EV secretion and uptake for fulfilling their biomedical potentials.

## Results

### Substrate stiffness regulates the biomolecular composition of MSC-EVs

**Fig. 1:**
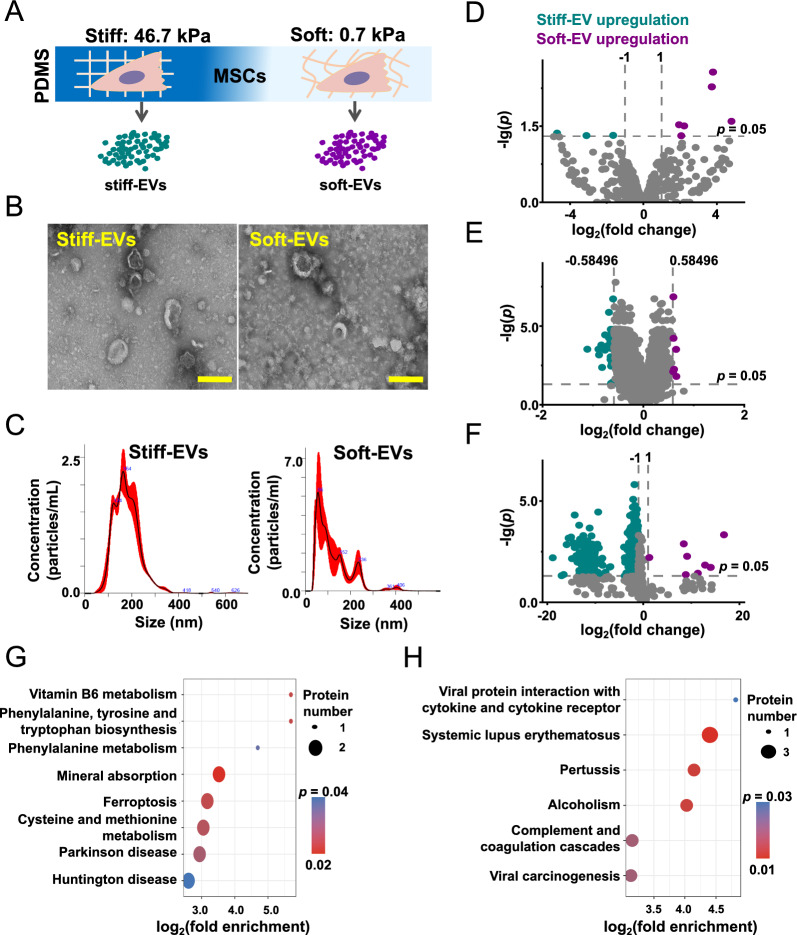
Profiling of substrate stiffness-induced MSC-EVs. **A**, Schematic representation of MSC-EVs acquisition induced by PDMS-prepared substrates with different stiffness (stiff-EVs represent EVs secreted by MSCs on stiff substrates; soft-EVs represent EVs secreted by MSCs on soft substrates). **B**, TEM images of stiff-EVs and soft-EVs. Scale bar: 200 nm. **C**, Characterization of particle sizes for stiff-EVs and soft-EVs measured by using nanoparticle tracking analysis (NTA). **D**–**F**, Volcano diagrams showing the differences in biomolecules of stiff-EVs and soft-EVs. **D**, miRNA (|log_2_(FC)|≥ 1; *p* ≤ 0.05 is defined as difference, *n* = 3); **E**, Protein (|log_2_(FC)|≥ 0.58; *p* ≤ 0.05 is defined as difference, *n* = 3); **F**, Lipid ((|log_2_(FC)|≥ 1; *p* ≤ 0.05 is defined as difference, *n* = 3). **G**–**H**, KEGG enrichment of proteins regulated by substrate stiffness in vesicles, **G**, up-regulated KEGG enrichment in stiff-EVs; **H**, up-regulated KEGG enrichment in soft-EVs (log_2_(FC) > 6; *p* < 0.05, *n* = 3)

Following our previous work [27], we prepared PDMS substrates of different stiffnesses (46.7 kPa defined as stiff and 0.7 kPa defined as soft) to represent a range of substrate stiffness that could be physiologically relevant for cell mechanosensing. Umbilical cord (UC) MSCs were incubated on the different substrates for 12 h, after which the medium was replaced with serum-free DMEM. After another 48 h of incubation, EVs were collected from the supernatant using ultracentrifugation [28]. We defined the EVs secreted by MSCs cultured on a stiff substrate as stiff-EVs, and those secreted on the soft substrate as soft-EVs (Fig. 1A). Transmission electron microscopy (TEM) showed that both soft- and stiff-substrate separated EVs had rounded and cup-shaped structures (Fig. 1B). To further determine the number of EVs, we analyzed EVs by nanoparticle tracking analysis (NTA). The NTA results supported the presence of heterogeneous EVs (Fig. 1C). We found that the soft substrate significantly enhanced the EV release compared to that in stiff group (Additional file 1: Fig. S1A). In addition, EVs collected from soft substrates have an average size of approximately 120.2 ± 75.1 nm, which is smaller than the average size of 179.6 ± 57.1 nm observed for EVs derived from stiff substrates. However, the difference did not reach statistical significance (Additional file 1: Fig. S1B). To further analyze the EV morphology, we performed a nano-flow cytometry analysis. Considering that the size range of exosomes is typically betweeen 30 and 180 nm, we chose 180 nm beads as reference for size detection (V-SSC) in nano-flow cytometry to enhance this observation [29–31]. By conducting single vesicle analysis, we observed that the majority of particles (> 75%) exhibited a particle size below 180 nm (Additional file 1: Fig. S2).

Subsequently, the miRNA, protein, and lipid characteristics of stiff-EVs and soft-EVs were profiled utilizing high-throughput sequencing, proteomics, and metabolic lipidomics. As signaling molecules carried by EVs, miRNAs play an important role in their functions [32]. High-throughput sequencing was used to identify miRNAs in stiff-EVs and soft-EVs. A total of 716 miRNAs were identified using the Illumina HiSeq2500 platform, of which 700 are already known and 16 are newly predicted (Additional file 2: Table S1). The data reveal that 9 miRNAs were significantly different between stiff-EVs and soft-EVs (Fig. 1D). Among them, three miRNAs were highly expressed in stiff-EVs and six miRNAs were highly expressed in soft-EVs.

To identify the proteins in EVs, proteomics was employed to characterize the proteins in stiff-EVs and soft-EVs. A total of 2944.0 quantifiable proteins were identified, of which 40 proteins were significantly different between stiff-EVs and soft-EVs (Fig. 1E, Additional file 3: Table S2). Among them, 33 proteins were highly expressed in stiff-EVs and seven proteins were highly expressed in soft-EVs. Lipids, as major components of EVs, were characterized using lipid metabolomics. A total of 1127 lipid metabolites were detected, of which 634 metabolites were significantly different between stiff-EVs and soft-EVs (Fig. 1F, Additional file 4: Table S3). Among these, 626 metabolites were highly expressed in stiff-EVs, and eight metabolites were highly expressed in soft-EVs.

These results suggest that substrate stiffness plays a significant role in regulating the biomolecular composition of EVs. An interesting observation from our data was that substrate stiffness drastically regulates MSC-EVs lipid and protein components. However, the regulation of miRNA components was not significant. The study employed multi-omics to provide a comprehensive profile of how substrate stiffness influences the biomolecular composition of EVs.

Functional analyses of EV proteins regulated by substrate stiffness were performed using KEGG enrichment. The data revealed that up-regulated proteins in stiff-EVs were mainly enriched in pathways related to systemic lupus erythematosus, pertussis, alcoholism, complement and coagulation cascades, viral oncogenesis, and interactions of viral proteins with cytokines and cytokine receptors (Fig. 1G). On the other hand, up-regulated proteins in soft-EVs were mainly enriched in pathways related to mineral absorption, vitamin B6 metabolism, phenylalanine, tyrosine and tryptophan biosynthesis, iron metabolism, cysteine and methionine metabolism, Parkinson's disease, phenylalanine metabolism, and Huntington's disease (Fig. 1H). The above results suggest that substrate stiffness can regulate MSC-EV biomolecule components and functions.

### Substrate stiffness regulates the synthesis and loading of biomolecules in MSC-EVs

Next, we sought to investigate the effect of substrate stiffness on the regulation of EV cargo sorting. Our preliminary work has shown that substrate stiffness plays a vital role in the regulation of cellular miRNA expression and sorting into EVs [28]. Therefore, we examined the regulation process of MSC-EVs protein and lipid by substrate stiffness. We cultured MSCs on substrates with varying stiffness for 48 h, and collected and characterized them using proteomics and lipid metabolomics.

**Fig. 2:**
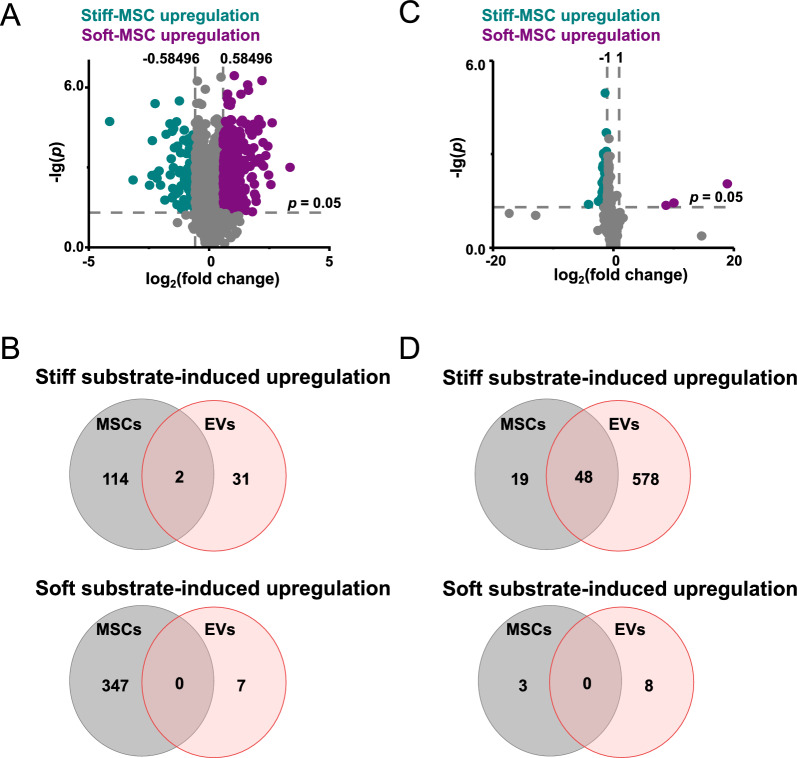
Substrate stiffness regulates the expression of biomolecules in MSCs. **A**, Volcano map demonstrating the regulation of MSCs proteins by substrate stiffness (|log2(FC)|≥ 0.58; p ≤ 0.05 is defined as difference, n = 3). **B**, Venn diagram of differential proteins regulated by substrate stiffness in MSCs (left) and MSC-EVs (right).** C**, Volcano map demonstrating the regulation of MSCs lipids by substrate stiffness ((|log2(FC)|≥ 1; p ≤ 0.05 is defined as a difference, n = 3).** D**, Venn diagram of differential lipids regulated by substrate stiffness in MSCs (left) and MSC-EVs (right)

A total of 4471 quantifiable proteins were identified, of which 463 proteins exhibited significant differences between MSCs cultured on stiff and soft substrates (Fig. 2A and Additional file 5: Table S4). Among these, 116 proteins were highly expressed in MSCs cultured on stiff substrates, while 347 proteins were highly expressed in MSCs cultured on soft substrates. Next, proteins regulated by substrate stiffness in cells and EVs were categorized by Venn diagrams. The results showed that only two of the 33 proteins (S100A10 and S100A11) upregulated in stiff-EVs were similarly upregulated in MSCs on stiff substrates (Fig. 2B). In contrast, the proteins upregulated in soft-EVs remained unchanged in MSCs cultured on soft substrate. These findings suggest that substrate stiffness primarily regulates the packaging of proteins into EVs.

Additionally, we detected a total of 1126 lipid metabolites, out of which 70 showed significant differences between MSCs on different substrates (Fig. 2C and Additional file 6: Table S5). Among them, 67 lipid metabolites were highly expressed in MSCs on stiff substrates, while 3 lipid metabolites were highly expressed in MSCs on soft substrates. Next, lipid metabolites regulated by substrate stiffness in cells and EVs were categorized by Venn diagrams. The results showed that 48 of the 626 lipid metabolites upregulated in stiff-EVs were similarly upregulated in MSCs on stiff substrates (Fig. 2D). Conversely, lipid metabolites upregulated in soft-EVs were unchanged in MSCs on soft substrates.

### Substrate stiffness regulates MSC lipid metabolic processes and protein membrane localization.

**Fig. 3:**
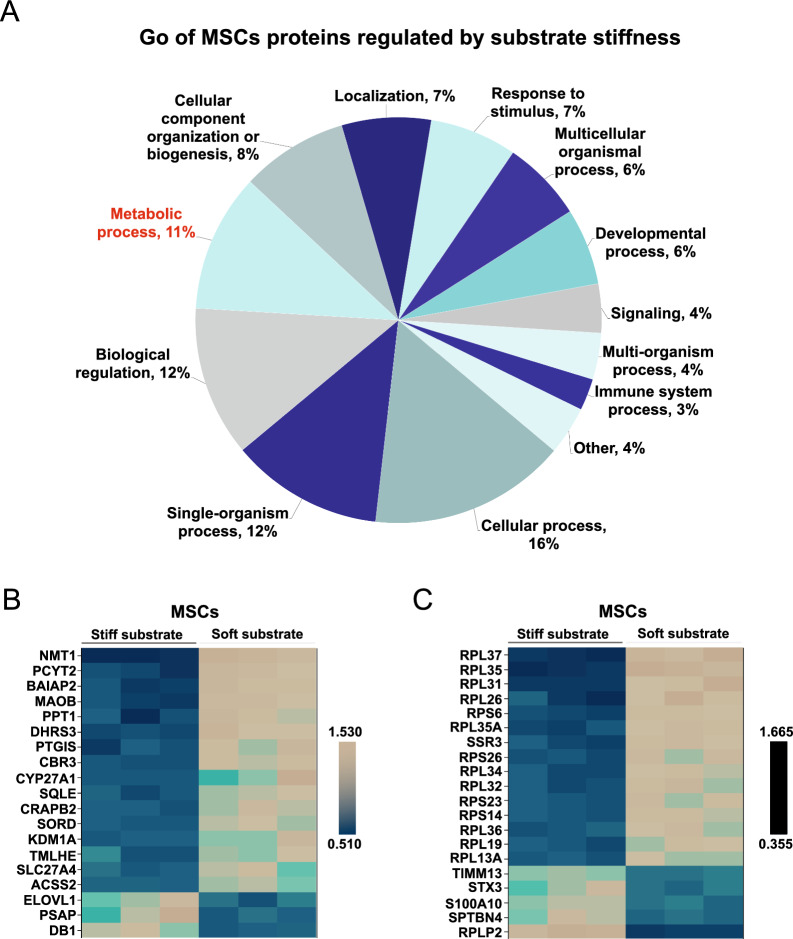
Substrate stiffness regulates cellular lipid metabolism of MSCs. **A**, GO analysis of MSCs cellular proteins regulated by substrate stiffness (*n* = 3). **B**, Heat map of proteins regulated by substrate stiffness associated with lipid metabolism and secondary metabolites (COG classification, *n* = 3). **C**, Heat map of proteins regulated by substrate stiffness and associated with protein membrane localization (GO enrichment, Top 20 with the minimum *p*, *n* = 3)

We have discovered notable variations in lipid metabolites between EVs and cells. To gain insight into how matrix stiffness affects EV components by modulating MSCs, we conducted a Gene Ontology (GO) analysis to examine the proteins influenced by substrate stiffness in MSCs. The results demonstrate that approximately 11% of regulated proteins of biological processes were associated with cellular metabolic processes (Fig. 3A). These findings strongly indicate that substrate stiffness has a significant impact on metabolic processes of MSCs. To further investigate the protein changes related to cellular lipid metabolism, we performed a COG/KOG analysis on the proteins regulated by substrate stiffness. The outcomes reveal that a total of 19 proteins, which are associated with lipids and secondary metabolites, were influenced by substrate stiffness (Additional file 1: Fig. S3). Moreover, the heatmap analysis demonstrates that a stiff substrate significantly upregulated the expression of proteins related to lipid transport and metabolism (ELOVL1, PSAP, DBI). On the other hand, a soft substrate significantly upregulated the expression of proteins related to secondary metabolites (NMT1, PCYT2, BAIAP2, MAOB, PPT1, DHRS3, PTGIS) and lipid metabolism (Fig. 3B).

Subsequently, we conducted GO enrichment analysis on differential proteins regulated by substrate stiffness. The results reveal that 44 regulated proteins were enriched in biological processes related to protein membrane localization (Additional file 1: Fig. S4). The heatmap analysis demonstrates that a stiff substrate significantly upregulates proteins associated with membrane localization, such as TIMM13, while a soft substrate significantly upregulate proteins associated with membrane localization, such as PRL37 (Fig. 3C). Protein membrane localization plays a crucial role in sorting cellular proteins into EVs [33–35]. Based on the above data, we hypothesize that substrate stiffnesses may affect the secretion of lipid components in MSC-EVs by regulating the cellular lipid metabolic process.

### MSC-EVs regulated by substrate stiffness affected macrophage phagocytosis.

**Fig. 4:**
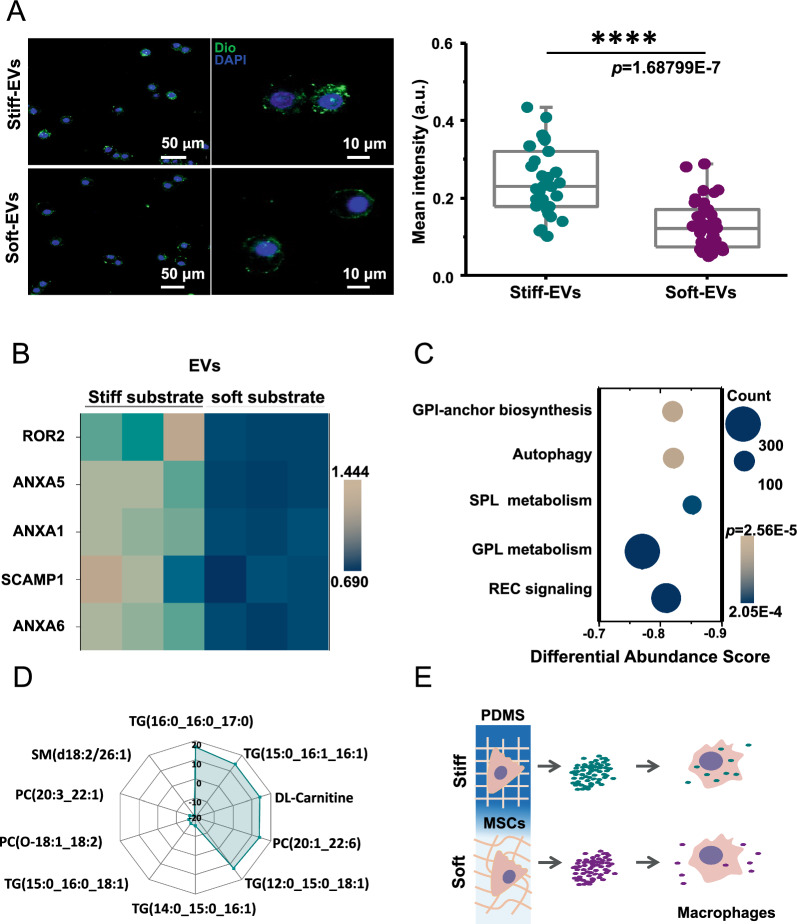
Substrate stiffness regulates the phagocytosis of MSC-EV in macrophages. **A**, Immunofluorescence assessment of macrophage phagocytic efficiency for stiff-EVs and soft-EVs. Left: fluorescence images (green for DiO-labeled EVs, blue for DAPI-labeled nucleus), Right: intracellular fluorescence intensity statistics (****p < 0.0001, n > 30). **B**, Heat map of proteins regulated by substrate stiffness and associated with vesicular transport (COG classification). **C**, Differential abundance (DA) scores for metabolites regulated by substrate stiffness (soft-EVs vs stiff-EVs Downgrade top5 with the minimum p, n = 3). **D**, Radar mapping of the top 10 metabolites with the greatest differences between soft-EVs and stiff-EVs (soft-EVs vs. stiff-EVs, Top 10 with the largest multiplicative differences, n = 3). E, The phagocytic behaviors in macrophages could vary depending on the types of EVs present

Our investigation focused on the analysis of differential proteins found in stiff-EVs and soft-EVs using COG classification. Our findings revealed that five of the proteins up-regulated in stiff-EVs were associated with vesicular transport (Additional file 1: Fig. S5). Based on this, we formulated a hypothesis that stiff-EVs and soft-EVs could exhibit heterogeneity in cellular uptake process. We chose macrophages as the targets of EVs because of their crucial role in maintaining overall health and defending against diseases. The EVs were labeled with DiO and co-incubated with RAW 264.7 cells, followed by nuclei labeling using DAPI. The cells were then visualized with confocal microscopy. Our results revealed that macrophages co-incubated with stiff-EVs exhibited higher fluorescence intensity (Fig. 4A), indicating that macrophages have a higher phagocytic efficiency for stiff-EVs compared to soft-EVs.

Through GO classification analysis, it was discovered that the miRNA molecules in stiff-EVs and soft-EVs did not affect genes related to EV uptake (Additional file 1: Fig. S6). Additionally, a heatmap demonstrated that proteins associated with vesicular transport were notably down-regulated in soft-EVs, which could explain the decreased efficiency of macrophage phagocytosis of soft-EVs (Fig. 4B). In terms of lipid metabolites, KEGG pathway analysis revealed that there were 111 metabolites enriched in autophagy-related pathways, with a negative differential abundance score (Fig. 4C, soft-EVs vs. stiff-EVs). Our study shows that there is a significant decrease in the amount of lipids in sof-EVs when compared to stiff-EVs. We used radar plots to demonstrate the difference in lipid fractions between the two types of EVs (Fig. 4D, soft-EVs vs. stif-EVs, Top 10). We believe that the lipid metabolite components present in the radargram could play a vital role in determining the efficiency of EV phagocytosis by macrophages. In summary, our results show that stiff-EVs and soft-EVs exhibit varying behaviors of phagocytosis in macrophages (Fig. 4E).

## Conclusions

We demonstrate that ECM stiffness has a notable effect on the components of MSC-EVs, which in turn regulates their behaviors. Specially, ECM stiffness affects the sorting of lipids and proteins into EVs, while miRNA regulation is not as significant. Our multi-omics analysis indicates that ECM stiffness can alter protein fractions in EVs by affecting the protein membrane localization. Interestingly, our current knowledge suggests that ECM stiffness has a considerable impact on the loading of lipid fractions into EVs. Besides, we have discovered that stiff-EVs are more effective in targeting macrophages than soft-EVs, due to regulated lipid metabolites in EVs through autophagy. Our study provides valuable insights into the modulation of EVs to deliver bioactive molecules and influence the fate of target cells, offering new opportunities for the development of designable therapeutic agents.

## Methods

### Cell culture

Mycoplasma-negative umbilical cord-derived mesenchymal stem cells (MSCs) were cultured in Dulbecco's Modified Eagle Medium (DMEM, Gibco, 11885092) supplemented with 10% fetal bovine serum (Gibco, 10099141), 100 U/mL penicillin, and 100 μg/mL streptomycin (Gibco, 15070063). Mycoplasma-negative RAW 264.7 cells, were cultured in RPMI-1640 (Sigma, R2405) supplemented with 10% FBS (Gibco, 10099141), 100 U/mL penicillin, and 100 μg/mL streptomycin (Gibco, 15070063). Both cell types were maintained at a temperature of 37 °C with 5% CO_2_. The RAW 264.7 cells (ATCC) were obtained from Central South University Cell Bank (Changsha, China).

### Preparation of PDMS substrate

PDMS substrates with different levels of stiffness were prepared according to a previous study [27]. The components A and B of SYLGARD (Sylgard 184; Dow Corning, Midland, MI, USA) were mixed at ratios of 50:1 and 100:1. The mixture was then spread onto cell culture vessels (10- or 15 cm-diameter dish) and cured at 70 °C overnight. A solution of Sulfo-SANPAH (0.1 mg mL^−1^, 22 589; Thermo Scientific, Waltham, MA, USA) was dropped onto the surface of the PDMS substrate and exposed to UV light for 10 min. After removing the Sulfo-SANPAH solution, it was further irradiated for 5 min. The PDMS substrate was then coated with collagen I (25 μg mL^−1^) after being washed twice with phosphate-buffered saline (PBS). The mechanical properties of the PDMS substrates were evaluated using a rotational rheometer (HAAKE MARS III; Thermo Scientific) in oscillation mode (0.1–10 Hz, 25 °C) with parallel plates as described by a previous study[27]. The data obtained were analyzed using RheoWin Data Manager software (Thermo Scientific).

### EVs collection

MSCs were incubated for 12 h on substrates with different stiffnesses, washed twice with PBS and replaced with serum-free medium. After another 48 h of incubation, the supernatant was collected. Then, the collected supernatants were subjected to centrifugation at 1500 g for 20 min at 4 °C to eliminate cell debris. Subsequently, the supernatants were filtered through a 0.22 μm filter (SLGP033RB, Millipore). The filtered supernatants were then subjected to centrifugation at 150,000 g for 2 h at 4 °C. The resulting EV pellet was resuspended in PBS for further use.

### Characterization of EVs

The size and number of EV particles were measured using Nanoflow (Apogee Flow Cytometer A50-Micro) following the instructions provided in the user manual. The data obtained from the EVs were collected and analyzed using Apogee Histogram software. The hydrodynamic size and concentration of samples were measured using nanoparticle tracking analysis (NTA) with Zeta View PMX 110 (Particle Metrix, Meerbusch, Germany) and corresponding software Zeta View 8.04.02.

### Transmission Electron Microscopy Imaging

The EVs dissolved in PBS were aspirated, and 10 μL of the solution (200 ng/μL) was added dropwise onto a copper mesh. The mixture was allowed to settle for 1 min, and any excess liquid was absorbed using filter paper. Subsequently, 10 μL of phosphotungstic acid was added dropwise onto the copper mesh and allowed to settle for 1 min, followed by absorption of any floating liquid using filter paper. The samples were then dried at room temperature for a few minutes. Electron microscopy imaging (HT-7700, Hitachi) was performed at 80 kV.

### Fluorescent Labeling with EVs

After DiO (1 μM) incubation with EVs (20 μg/μL) for 30 min, the EVs were collected by ultracentrifugation at 12,000 × g for 15 min. Then, the DiO-labelled EV pellet was resuspended in PBS buffer. The concentration of DiO-labelled EV particles was characterized using Nanoflow and adjusted to ensure consistency before being used for subsequent EV uptake experiments.

### RAW 264.7 cells imaging

RAW 264.7 cells were plated at 60%-70% confluency for 24 h in 35-mm culture dishes. Then, cultured cells were washed with PBS (pH 7.4) two times before imaging. After incubation with DiO-labelled EVs (50 μg/mL) from different substrates for 6 h, the medium was removed. The treated RAW 264.7 cells were washed with PBS. Before the imaging, the cells were stained with DAPI. All images were obtained using a laser confocal microscope (Leica TCS SP8). Wavelength sets were 488 nm excitation (Ex)/510–650 nm emission (Em) for DiO-labeled EVs. The cellular uptake of EVs was quantified by determining the MFI value. Quantization by plots was accomplished using the software package provided by Leica Instrument. Each of the experiments was performed at least 3 times.

### RNA-seq data analysis

*High-throughput sequencing of miRNA in exosomes.* Total RNA was extracted and purified from EVs using miRNeasy Serum/Plasma Advanced Kit (Qiagen, cat. No. 217204) according to the kit instruction. RNA concentration and purity were evaluated using the RNA Nano 6000 Assay Kit of the Agilent Bioanalyzer 2100 System (Agilent Technologies, CA, USA). For small RNA libraries, a total amount of 3.0 ng RNA per sample was used as input material for the RNA sample preparations. Sequencing libraries were generated using QIAseq miRNA Library Kit (Qiagen, Frederick, MD) following the manufacturer’s recommendations, and index codes were added to attribute sequences to each sample. Reverse transcription (RT) primers with unique molecular indices (UMIs) were introduced to analyze the quantification of miRNA expressions during cDNA synthesis and PCR amplification. At last, library quality was assessed on the Agilent Bioanalyzer 2100 and qPCR. The clustering of the index-coded samples was performed on the acBot Cluster Generation System using TruSeq PE Cluster Kitv3-cBot-HS (Illumina, San Diego, CA, USA) according to the manufacturer’s instructions. After cluster generation, the library preparations were sequenced on an Illumina HiSeq2500 platform, and paired-end reads were generated at EchoBiotech Co. Ltd., Beijing, P. R. China.

*Differential expression analysis.* Bowtie tools were used to align the clean reads with various databases including Silva, GtRNAdb, Rfam, and Repbase. This alignment helped filter out ribosomal RNAs (rRNAs), transfer RNAs (tRNAs), small nuclear RNAs (snRNAs), small nucleolar RNAs (snoRNAs), other non-coding RNAs (ncRNAs), and repetitive sequences. The remaining reads were then used to detect known miRNAs and predict novel miRNAs by comparing them with the genome and known miRNAs from miRBase. |log_2_(FC)|≥ 1 and p ≤ 0.05 were defined as differentially expressed. Target gene of differentially expressed miRNA prediction was performed using miRanda[36] and RNAhybrid[37] tools. For novel miRNA secondary structure prediction, the Randfold software was utilized.

*Gene Ontology (GO) enrichment analysis.* To analyze the differentially expressed genes (DEGs), GO enrichment analysis was conducted using the topGO R package. High-throughput sequencing and data analysis of miRNAs was done by Biomarker Inc. Three independent samples were measured in each group.

### Bioinformatics analysis of proteins

*Protein Pretreatment*. The MSCs and EVs samples were subjected to sonication three times on ice using a high-intensity ultrasonic processor (Scientz) in lysis buffer containing 8 M urea and 1% Protease Inhibitor Cocktail. After sonication, the remaining debris was eliminated by centrifugation at 12,000 g at 4 °C for 10 min. Subsequently, the supernatant was collected, and the protein concentration was determined using a BCA kit following the manufacturer's instructions. After extraction, the protein solution was treated by trypsin digestion, TMT/iTRAQ Labeling, HPLC Fractionation, and LC–MS/MS Analysis.

*Differential expression analysis.* A total of 5130.0 proteins were identified, of which 4471.0 were quantifiable (quantifiable proteins indicate that quantitative information is available for at least one of the comparator groups) after searching the library of theoretical protein data using mass spectrometry secondary spectra. |log_2_(FC)|≥ 0.58 and p ≤ 0.05 were defined as differentially expressed.

*Clusters of Orthologous Groups (COG) classification.* To gain a comprehensive understanding of the identified and quantified proteins in our data, we analyzed the differentially expressed protein functions and characteristics using GO. The GO annotation proteome was obtained from the UniProt-GOA database (http://www.ebi.ac.uk/GOA/). Furthermore, we performed COG functional classification of the differentially expressed proteins by comparing them to a database.

*Enrichment of GO analysis.* To assess the enrichment of differentially expressed proteins within this category, a two-tailed Fisher's exact test was employed. GO terms with a corrected p < 0.05 were considered significant in terms of enrichment. Proteomics detection and data analysis were completed by PTM Bio Inc. Three independent samples were measured in each group.

### Bioinformatics analysis of lipids

*Lipid pretreatment.* The MSCs and EVs samples were retrieved from a − 80 °C refrigerator and thawed on ice. 1 mL of extraction solvent (MTBE: MeOH = 3:1, v/v), containing an internal standard mixture, was added to the sample. The mixture was vortexed for 15 min to ensure proper extraction. 200 μL of water was added to the mixture. The mixture was vortexed for an additional 1 min. Centrifugation was performed at 12,000 rpm for 10 min. 500 μL of the upper organic layer was carefully collected. The collected organic layer was evaporated using a vacuum concentrator. The resulting dry extract was reconstituted using 200 μL of mobile phase B. The reconstituted extract was then ready for LC–MS/MS analysis.

*Differential metabolites selected.* Lipid metabolites were qualitatively analysed based on the retention time RT (Retention time) and secondary spectral data of the detected substances, as well as the self-constructed targeted specimen database MWDB (metware database). Metabolite quantification was done using multiple reaction monitoring analysis. |log_2_(FC)|≥ 1 and VIP ≥ 1were defined as differential metabolites.

*KEGG annotation and enrichment analysis.* The identified differential metabolites were annotated using the KEGG Compound database (http://www.kegg.jp/kegg/compound/). Subsequently, the annotated metabolites were mapped to the KEGG Pathway database (http://www.kegg.jp/kegg/pathway.html). To identify significantly enriched pathways, a hypergeometric test was performed using the p for a given list of metabolites.

Lipoomics detection and data analysis were completed by PTM Bio Inc. Three independent samples were measured in each group.

### Statistical analysis

The data are expressed as the mean ± SD. Differences among groups were determined using analysis of variance two-factor for repeated measurements.

### Supplementary Information

**Additional file 1:** **Figure S1.** The concentrations and average particle sizes of EVs obtained by NTA. **Figure S2.** Characterization of EVs using nano-flow cytometry. **A**, **B** The SSC distribution histograms of EVs. **Figure S3.** COG/KOG classification of UMSC cellular proteins regulated by substrate stiffness. **Figure S4.** Substrate stiffness regulates the localization of proteins within the UMSCs. GO enrichment of UMSC proteins regulated by substrate stiffness. COG/KOG classification of UMSC proteins regulated by substrate stiffness. **Figure S5.** COG classification of down-regulated proteins in soft-EVs. **Figure S6.** GO classification of differentially expressed miRNA-targeting genes.

**Additional file 2.** Identification and quantitative information of miRNAs in stiff-EVs and soft-EVs.

**Additional file 3.** Identification and quantitative information of proteomics in stiff-EVs and soft-EVs.

**Additional file 4.** Identification and quantitative information of lipidomics in stiff-EVs and soft-EVs.

**Additional file 5.** Identification and quantitative information of proteomics in MSCs cultured on stiff and soft substrates.

**Additional file 6.** Identification and quantitative information of lipidomics in MSCs cultured on stiff and soft substrates.

## Data Availability

All the data supporting the conclusions are available in the main text and Additional file information.

## Abbreviations

EV: Extracellular vesicle
MSC: Mesenchymal stem cell
ECM: Extracellular matrix
PDMS: Polydimethylsiloxane
DMEM: Dulbecco's Modified Eagle Medium
KEGG: Kyoto Encyclopedia of Genes and Genomes
GO: Gene Ontology
COG: Clusters of orthologous groups of proteins
KOG: Clusters of Orthologous Groups for Eukaryotic Complete Genomes
ELOVL1: Elongase of very long chain fatty acid 1
PSAP: Prosaposin
DBI: Diazepam binding inhibitor
NMT1: N-myristoyltransferase 1
PCYT2: Phosphate cytidylyltransferase 2
BAIAP2: Brain-specific angiogenesis inhibitor 1-associated protein 2
MAOB: Monoamine oxidase B
PPT1: Preprotachykinin 1
DHRS3: Dehydrogenase/reductase SDR family member 3
PTGIS: Prostaglandin I Synthase
TIMM13: Translocase of inner mitochondrial membrane 13
PRL37: Prolactin 37
REC: Retrograde endo cannabinoid
GPL: Glycerophospholipid
SPL: Sphingolipid
GPI: Glycosylphosphatidylinositol
