# Supplementary material for: ECM stiffness affects cargo sorting into MSC-EVs to regulate their secretion and uptake behaviors
Source: J Nanobiotechnology. 2024 Mar 21;22:124. doi: 10.1186/s12951-024-02411-w (PMC10956366; doi:10.1186/s12951-024-02411-w)
Supplement: Supplementary file 1 — Additional file 1: Figure S1. The concentrations and average particle sizes of EVs obtained by NTA. Figure S2. Characterization of EVs using nano-flow cytometry. A, B The SSC distribution histograms of EVs. Figure S3. COG/KOG classification of UMSC cellular proteins regulated by substrate stiffness. Figure S4. Substrate stiffness regulates the localization of proteins within the UMSCs. GO enrichment of UMSC proteins regulated by substrate stiffness. COG/KOG classification of UMSC proteins regulated by substrate stiffness. Figure S5. COG classification of down-regulated proteins in soft-EVs. Figure S6. GO classification of differentially expressed miRNA-targeting genes. [file 12951_2024_2411_MOESM1_ESM.docx]

Supporting Information

**ECM stiffness affects cargo sorting into MSC-EVs to regulate their secretion and delivery**

Zhixiao Liu^1†^, Yingying Liu^2†^, Yu Li^3,4†^, Sha Xu^3,4†^, Yang Wang^5^, Yuruchen Zhu^6^, Chu Jiang^7^, Kaizhe Wang^8*^, Yinan Zhang^7*^, Yue Wang^3,4*^

^1^Department of Histology and Embryology, College of Basic Medicine, Naval Medical University, Shanghai, 200433, China

^2^School of Chemistry and Chemical Engineering, Center for Transformative Molecules, Zhangjiang Institute for Advanced Study and National Center for Translational Medicine (Shanghai), Shanghai Jiao Tong University, Shanghai 200240, China

^3^Stem Cell and Regeneration Medicine Institute, Research Center of Translational Medicine, Naval Medical University, Shanghai, 200433, China

^4^Shanghai Institute of Stem Cell Research and Clinical Translation, Shanghai 200120, China

^5^Shanghai General Hospital of Nanjing Medical University, Shanghai, 200086, China

^6^College of Basic Medicine, Naval Medical University, Shanghai, 200433, China

^7^School of Chemical Science and Engineering, Tongji University, Shanghai, 200092, China

^8^Ningbo Key Laboratory of Biomedical Imaging Probe Materials and Technology, Ningbo Cixi Institute of BioMedical Engineering, Ningbo Institute of Materials Technology and Engineering, Chinese Academy of Sciences, Ningbo, 315300, China

*Correspondence:

Kaizhe Wang

[wangkaizhe@nimte.ac.cn](mailto:wangkaizhe@nimte.ac.cn)

Yinan Zhang

[yinan_zhang@tongji.edu.cn](mailto:yinan_zhang@tongji.edu.cn)

Yue Wang

[wangyuesmmu@163.com](mailto:wangyuesmmu@163.com)

^†^These authors contributed equally to this work.


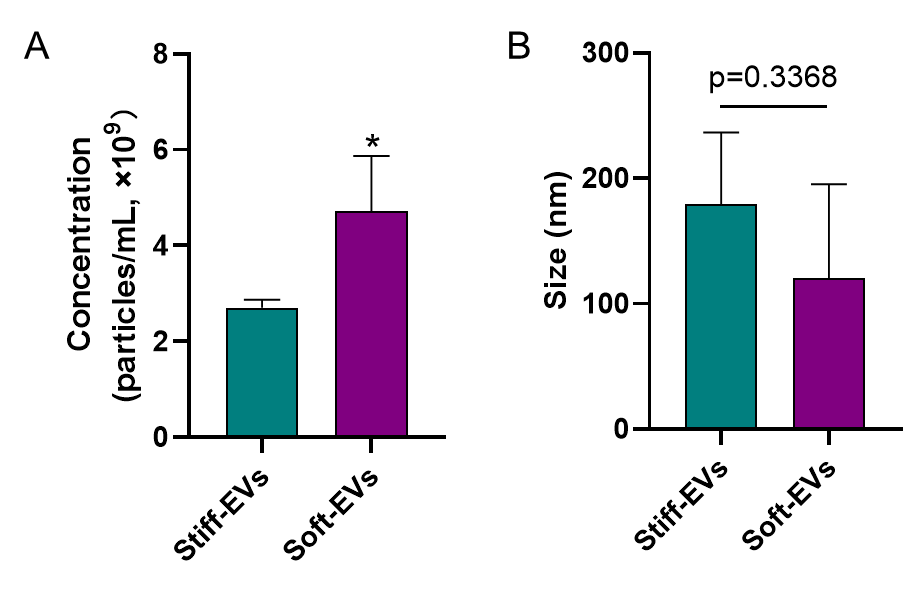


**Figure S1**. The concentrations and average particle sizes of EVs obtained by NTA.


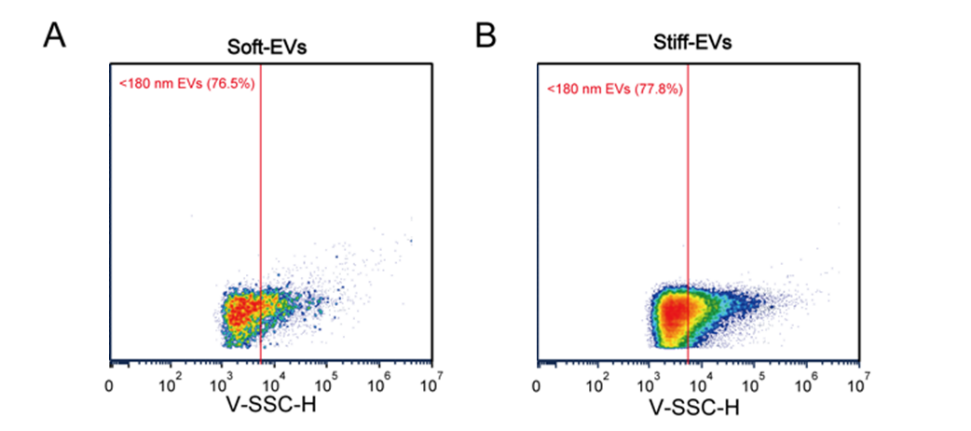


**Figure S2.** Characterization of EVs using nano-flow cytometry. (**A, B**) The SSC distribution histograms of EVs.


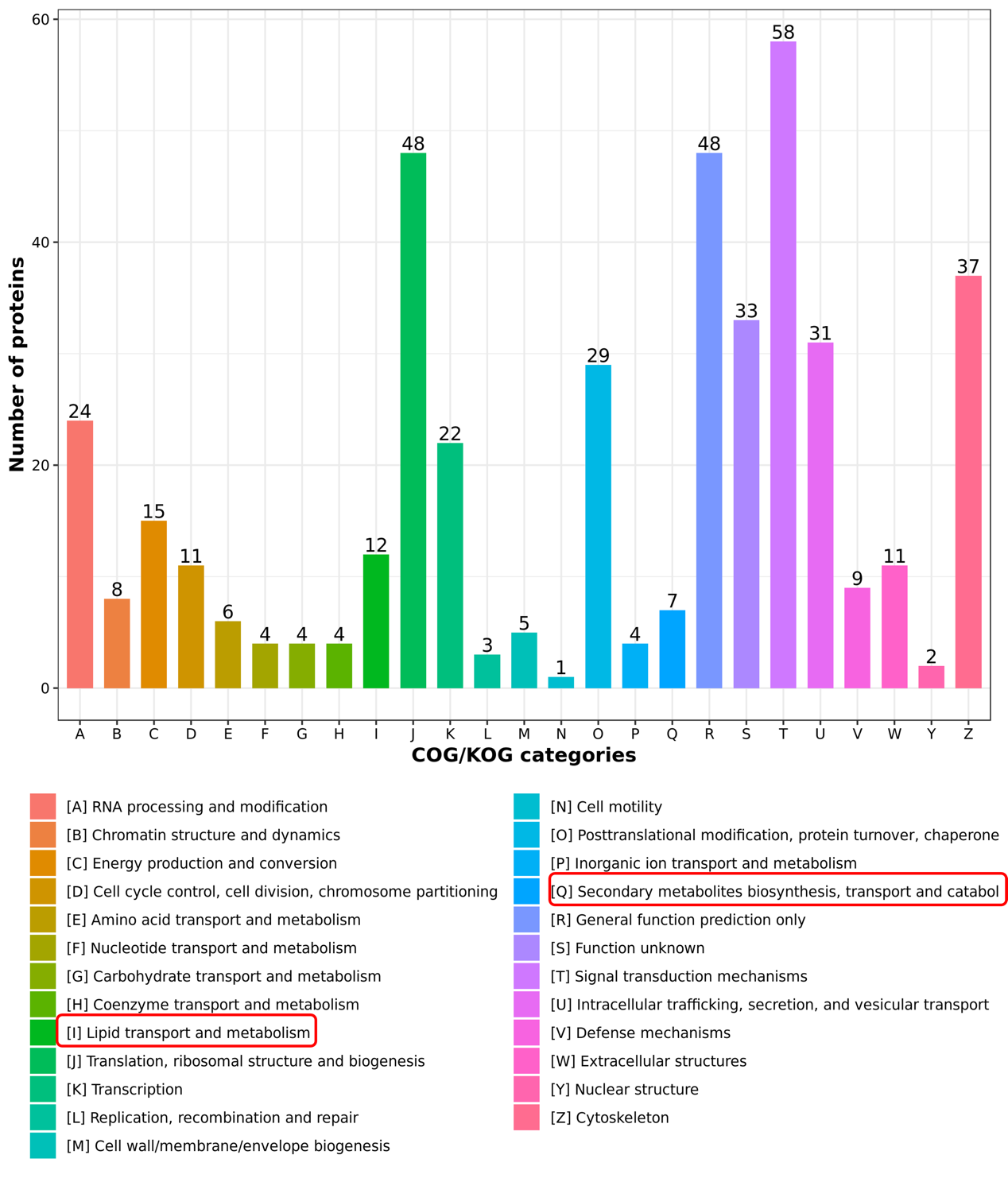


**Figure S3**. COG/KOG classification of UMSC cellular proteins regulated by substrate stiffness.


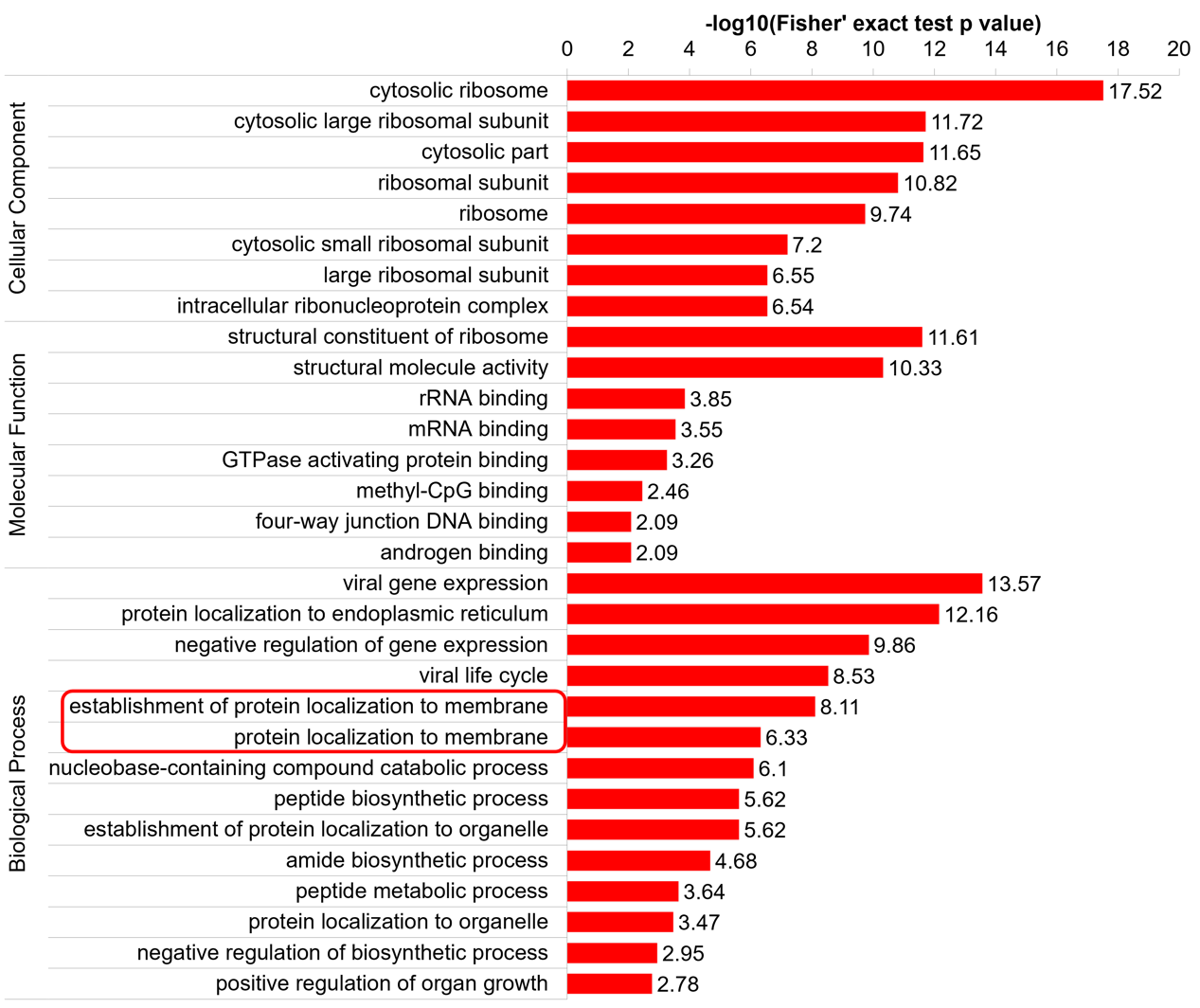


**Figure S4**. Substrate stiffness regulates the localization of proteins within the UMSCs. GO enrichment of UMSC proteins regulated by substrate stiffness. COG/KOG classification of UMSC proteins regulated by substrate stiffness.


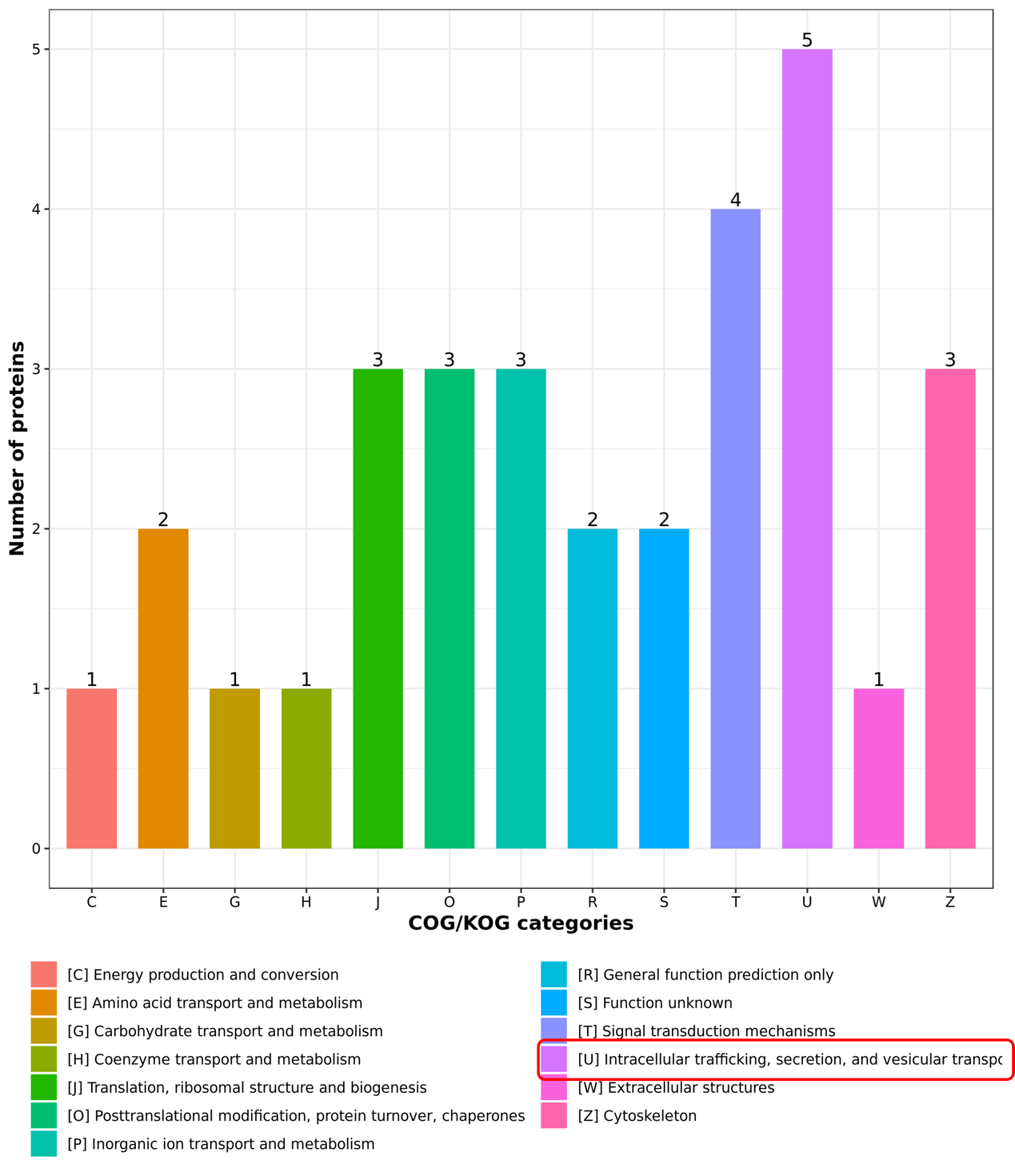


**Figure S5**. COG classification of down-regulated proteins in soft-EVs.


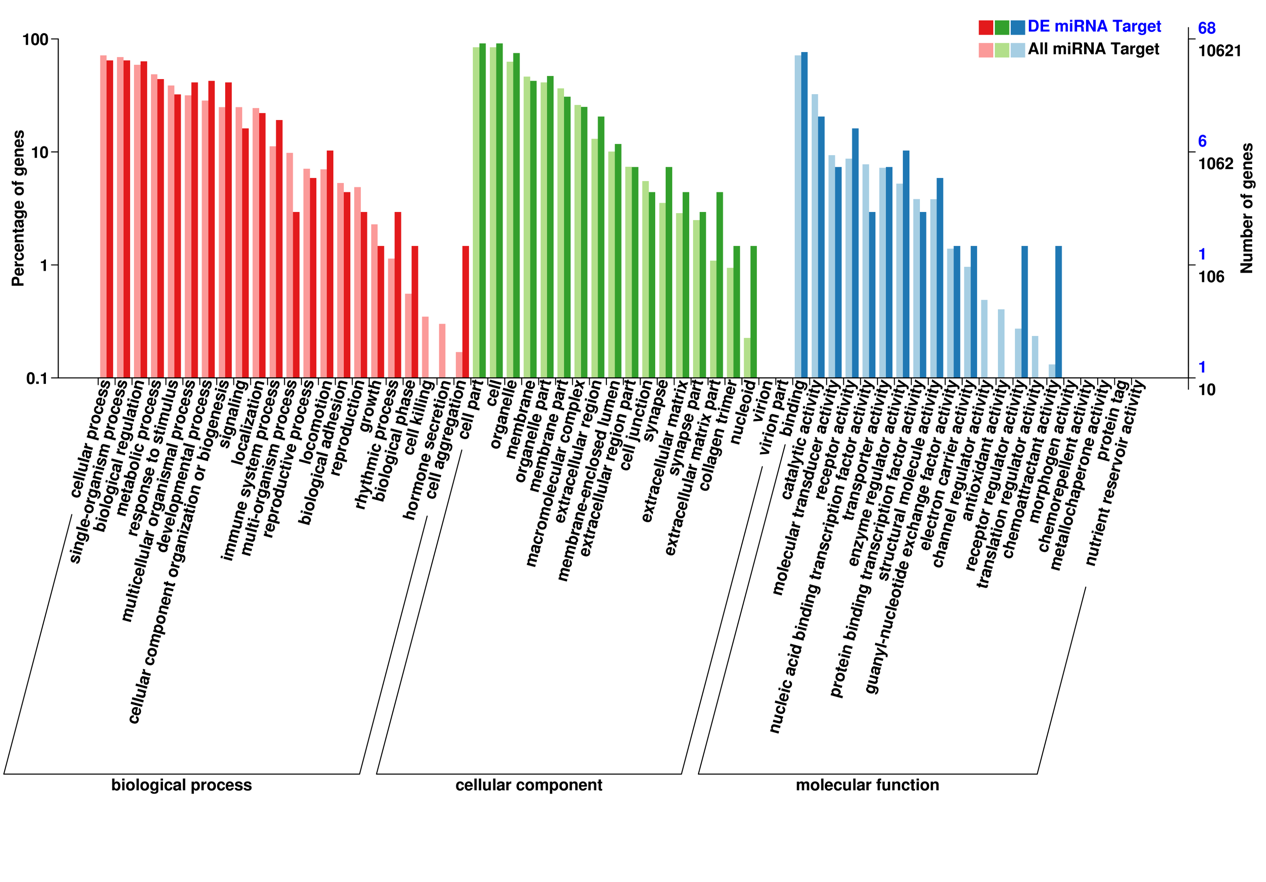


**Figure S6**. GO classification of differentially expressed miRNA-targeting genes.
